# Crash counterpart vehicles play important role in on pre-hospital mortality: A Study Based on ‎an eight-year period, 2006-2014‎ 

**Published:** 2019-08

**Authors:** Homayoun Sadeghi-Bazargani, Bahram Samadirad, Nasrin Shahedifar, Mina Golestani

**Affiliations:** ^*a*^Road Traffic Injury Research Center, Statistics and Epidemiology Department, Tabriz University of Medical Sciences, Tabriz.; ^*b*^Legal Medicine Research Center, Legal Medicine Organization, Tehran, Iran.

## Abstract

**Background:**

To study the association between crash counterpart vehicles and pre-hospital car-users fatalities ‎during eight years, in East Azerbaijan, Iran. ‎

**Methods:**

The data related to 3051 car user road traffic fatalities (CURTFs) were registered in East ‎Azerbaijan forensic medicine organization database, Iran, during 2006-2014. Data analysis was ‎run using Stata 13 statistical software package. Descriptive statistics (Pvalue <0.05) and ‎inferential statistical methods such as Chi-squared test and multivariate logistic regression with ‎P <0.1 were applied. ‎

**Results:**

3051 (39%) of 7818 road traffic injury death were car users of whom 71% were male (mean age ‎of 36.7±18.5 years). In assessing the role of type of crash counterpart vehicle on pre-hospital ‎mortality, considering the other cars to be the reference group for comparison, deceased victims ‎were 1.83 times more likely to die before hospital when the counterpart vehicle was a truck and ‎‎1.66 times more for buses. ‎

**Conclusions:**

It seems necessary to identify those risk factors making crash counterpart vehicles have ‎significant role on pre-hospital mortality

**Keywords:**

Car Users, Crash Counterpart Vehicle, Mortality, Road Traffic Accidents, Epidemiology

